# Ultrarapid cryo-arrest of living cells on a microscope enables multiscale imaging of out-of-equilibrium molecular patterns

**DOI:** 10.1126/sciadv.abk0882

**Published:** 2021-12-10

**Authors:** Jan Huebinger, Hernan Grecco, Martín E. Masip, Jens Christmann, Günter R. Fuhr, Philippe I. H. Bastiaens

**Affiliations:** 1 Department of Systemic Cell Biology, Max Planck Institute of Molecular Physiology, Otto-Hahn-Str.11, 44227 Dortmund, Germany.; 2 Department of Physics, FCEN, University of Buenos Aires and IFIBA, CONICET, Buenos Aires, Argentina.; 3 Fraunhofer Institute for Biomedical Engineering, Joseph-von-Fraunhofer-Weg 1, 66280 Sulzbach, Germany.; 4 Faculty of Chemistry and Chemical Biology, TU Dortmund University, Otto-Hahn-Str. 6, 44227 Dortmund, Germany.

## Abstract

Imaging molecular patterns in cells by fluorescence micro- or nanoscopy has the potential to relate collective molecular behavior to cellular function. However, spatial and spectroscopic resolution is fundamentally limited by motional blur caused by finite photon fluxes and photobleaching. At physiological temperatures, photochemical reactivity does not only limit imaging at multiple scales but is also toxic to biochemical reactions that maintain cellular organization. Here, we present cryoprotectant-free ultrarapid cryo-arrest directly on a multimodal fluorescence microscope that preserves the out-of-equilibrium molecular organization of living cells. This allows the imaging of dynamic processes before cryo-arrest in combination with precise molecular pattern determination at multiple scales within the same cells under cryo-arrest. We both experimentally and theoretically show that ultrarapid cryo-arrest overcomes the fundamental resolution barrier imposed by motional blur and photochemical reactivity, enabling observation of native molecular distributions and reaction patterns that are not resolvable at physiological temperatures.

## Teaser

Fluorescence imaging under cryo-arrest allows resolution of dynamic signaling system features that are otherwise obscured.

## INTRODUCTION

Cellular functionality emerges at the micrometer scale from the collective, dynamic behavior of nanometer-sized molecules, which is maintained out of equilibrium by chemical energy conversion (*1*–*4*). The resulting molecular patterns that manifest at multiple spatial and temporal scales can be assessed at the micrometer scale by fluorescence microscopy and down to the nanoscale by methods such as single-molecule localization microscopy (SMLM) (*5*), nanoscopy with minimal photon flux (MINFLUX) (*6*), temporal fluorescence fluctuation approaches such as superresolution radial fluctuation (SRRF) (*7*), or coordinate-targeted nanoscopy such as stimulated emission depletion (STED) nanoscopy (*8*, *9*). In coordinate-targeted nanoscopy, fluorescence intensity images are directly related to molecular density, enabling the acquisition of complete molecular patterns. Adding a spectroscopic dimension to the readout of photons in micro- or nanoscopy, such as polarization (*10*, *11*) or excited-state lifetime (*12*, *13*), allows the quantitative determination of molecular reaction states within spatially resolved volume elements.

However, spatial and spectroscopic resolution is fundamentally limited by the number of collected photons because of the Poisson statistics of quantum interactions (*6*, *14*, *15*). The total amount of collectible photons is also limited by excited-state reactions, which lead to photobleaching of the fluorophores (*16*) and additionally result in phototoxicity, reducing biological accuracy of the measurement (*17*). The number of collectible photons per time is restricted by saturable cycling of fluorophores between the ground and excited state. This fundamental limit is determined by molecular cross section, fluorescence quantum yield, and excited-state lifetime of the fluorophores (*16*). Thus, a minimally attainable exposure time that is independent of excitation energy exists, which determines the amount of collected photons and thereby the signal-to-noise ratio to achieve a particular resolution. Furthermore, during this minimal attainable exposure time, Brownian or active motion blurs the localization of fluorescent structures and thereby again imposes a limit to resolution. This motional blur is further enhanced by methodological constrains that prolong exposure times, such as laser scanning in coordinate-targeted nanoscopy or switching of fluorophores in SMLM or MINFLUX (*9*). The full potential of fluorescence micro- or nanoscopy can therefore be exploited on immobile or very slowly moving macromolecular structures in living cells such as nuclear pores or stable parts of the cytoskeleton (*18*). However, many molecular patterns in living cells are time-varying reaction-diffusion processes that enable the adaptive behavior of cells to a changing environment (*4*, *19*). Because of the constraints set by motional blur and photobleaching, these transient states remain largely inaccessible.

A workaround to reduce molecular motion is chemical fixation of cells. Subsequent embedding in antifade media reduces the amount of singlet oxygen and, thus, the rate of photobleaching (*20*), which however remains a major limitation for photon collection in fluorescence microscopy (*9*, *16*, *17*). However, even more problematic is that the fixation process takes at least minutes, is incomplete (*21*, *22*), and inherently changes the sample by cross-linking, denaturation, and loss of material (*23*–*25*). Thus, the dynamic, out-of-equilibrium molecular patterns cannot be faithfully captured.

On the other hand, cells can be physically fixed by rapidly cooling the sample to a temperature where it becomes a solid or reaches very high viscosity (*24*, *26*). Yet, living cells have to be cooled extremely fast below the glass transition temperature of ≈−136°C to avoid mechanical destruction of cellular structures by ice crystals (*26*) as well as denaturation of lipid membranes and proteins (*27*). However, if cooling is rapid enough, water molecules cannot reach thermodynamic equilibrium and become locked in a liquid-like configuration (*28*), enabling the preservation of cellular out-of-equilibrium macromolecular patterns at low temperature. To capture native out-of-equilibrium molecular patterns at multiple scales under enabling photophysical as well as photochemical conditions (*29*), we developed cryoprotectant-free ultrarapid cryo-arrest directly during microscopic observation of living cells.

## RESULTS

### Ultrarapid cryo-arrest on a microscope

**Fig. 1.**
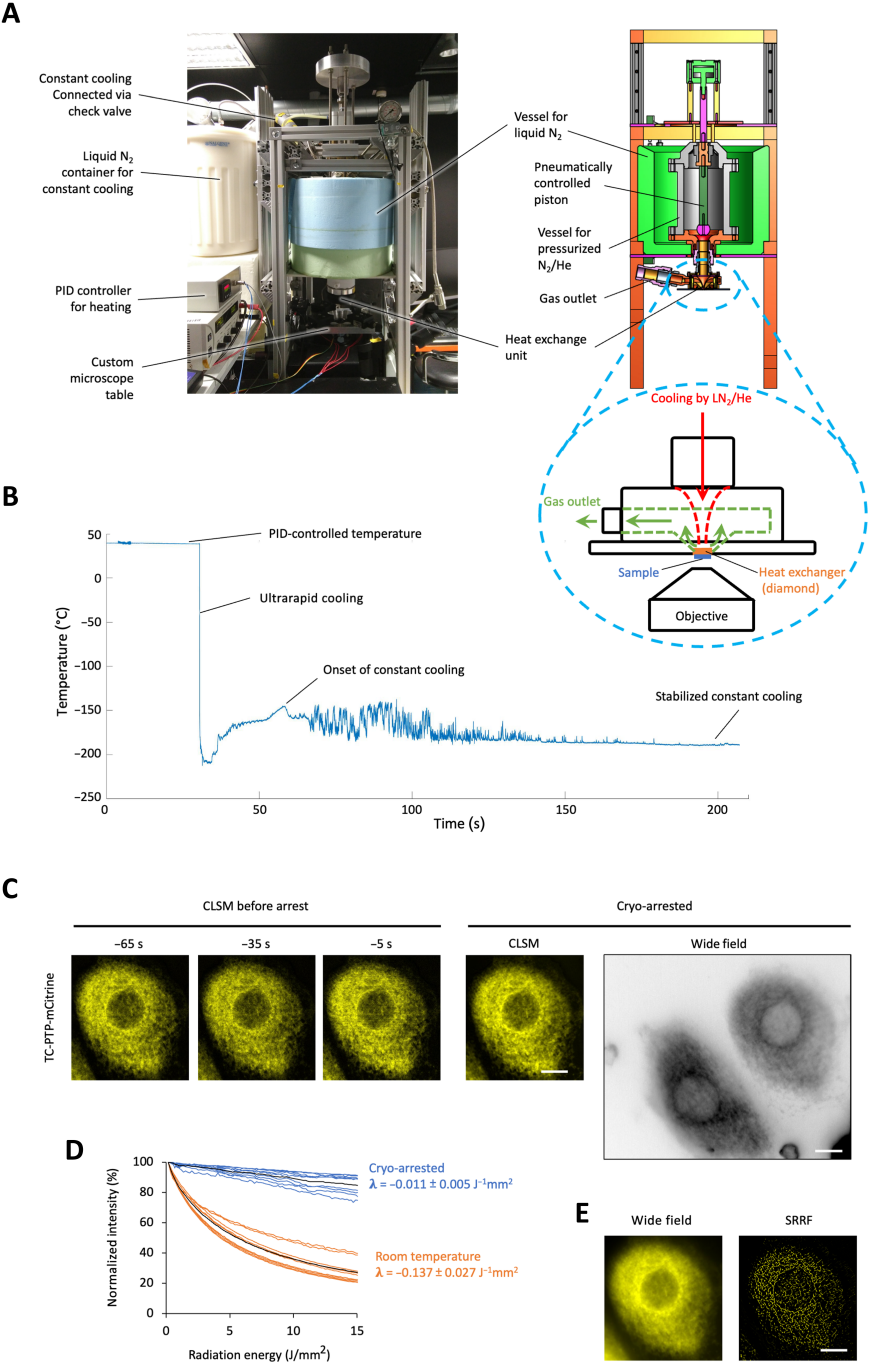
Ultrarapid cryo-arrest microscopy. (**A**) Photograph and schematic of ultrarapid cooling device. Cyan dashed circle: heat exchanger unit; red arrow: flow of liquid nitrogen (LN2) with gaseous He toward diamond heat exchanger; green arrows: expanded gas outflow. The whole cooling device is lowered above an epifluorescence microscope objective. (**B**) Measured temperature course (50-μm constantan-copper thermocouple in 100-μm aqueous sample). (**C**) CLSM of TC-PTP-mCitrine in a MCF7 cell before (at indicated time, 3.7-s scan time) and during cryo-arrest (10 frames, 37-s scan time). Right: Corresponding wide-field fluorescence image shown in inverted gray scale to visualize dim extensions of the endoplasmic reticulum. (**D**) mCitrine photobleaching at room temperature (orange; *N* = 9) and under cryo-arrest (blue; *N* = 9); 100 wide-field frames. λ: photobleaching rates (mean ± SD). (**E**) Sum (left) and SRRF reconstruction (right) from 100 wide-field frames under cryo-arrest. Scale bars, 10 μm.

To achieve the fastest possible cooling rates to arrest native molecular patterns in mammalian cells, we designed a setup where a sample on a diamond heat exchanger was rapidly cooled by a burst of pressurized liquid nitrogen (LN_2_: −196°C, 50 bars) on the opposite side of the diamond. The ensuing high flow of coolant suppresses film boiling, resulting in effective convective cooling of the diamond that rapidly extracts heat from the sample through its high thermal conductivity [single-crystal chemical vapor deposition (CVD) diamond: >2200 Wm^−1^ K^−1^] (*30*). A microscopy glass coverslip (∅ 4 mm) containing the sample was therefore adhered directly to the heat exchanger (∅ 5 mm) opposite to the high–numerical aperture (NA) air objective (NA: 0.95) of an epifluorescence microscope (Fig. 1A), which allows continuous imaging of the sample before and during cryo-arrest. To attain high-pressure release, LN_2_ and pressurized gaseous helium (He; 50 bars) were subsequently injected in a stainless steel vessel that was cooled by LN_2_ from the outside. An electronically triggered pneumatically driven piston in the vessel functioned as a fast-opening valve to release the pressurized mixture toward the heat exchanger as a sudden burst at a selected time point during the measurement (Fig. 1A). The device was further equipped with an electric heating to regulate temperature before the arrest and a check valve that automatically initiates a constant supply of cold nitrogen during cryo-arrest to maintain the temperature close to −196°C (Fig. 1, A and B).

We measured the cooling performance with a 50-μm thermocouple in a 100-μm-thick aqueous sample (thermal conductivity: <0.6 Wm^−1^ K^−1^). In the critical temperature range between 0° and −130°C, where ice crystallization can occur, cooling rates approached the theoretical thermal diffusivity–limited rate of about 13,000°C/s (fig. S1, A and B). This indicates that thinner samples, such as 10- to 15-μm-thick samples containing adherent mammalian cells, can reach cooling speeds of up to 200,000°C/s (fig. S1B). Wide-field fluorescence imaging before and during cryo-arrest of enhanced green fluorescent protein (EGFP)–expressing adhered HeLa cells indeed showed that cellular morphology was preserved, exhibiting no detectable ice crystals, which would result in fluorescence-free areas (*31*) inside of the cells (fig. S1C). This was further confirmed by the ultrarapid cooling of a 15-μm-thick rhodamine 6G solution, where microscopic ice crystals only emerged as fluorescence-free areas after subsequent slow rewarming (fig. S1D). Some fracturing after cryo-fixation did however occur, which can be explained by expansion of the solidified aqueous solution at −196°C (~1.8% in each dimension) (*32*, *33*).

To first investigate whether the intricate subcellular organization of mammalian cells was preserved upon ultrarapid cryo-arrest, the dynamically maintained morphology of the endoplasmic reticulum (ER) (*34*) was imaged before and under cryo-arrest by multiple fluorescence microscopy modalities. To label ER membranes, the ER-bound T cell protein tyrosine phosphatase (TC-PTP) was fused to the fluorescent protein mCitrine (TC-PTP-mCitrine) (*35*) and stably expressed in MCF7 cells. Confocal laser scanning microscopy (CLSM) and wide-field fluorescence imaging before and during cryo-arrest showed that the fenestrated reticular structure of the ER that extends into the periphery of adherent cells was fully preserved after ultrarapid cryo-arrest (Fig. 1C and fig. S2, A and B). A variable level of fractures occurred in the surrounding medium, infrequently extending into cells. However, those cells that were altered could easily be identified by comparison with the images of the living cells obtained before cryo-arrest (fig. S2, C and D). The largely increased photostability of mCitrine at cryo-temperatures (Fig. 1D) enabled the subsequent acquisition of 100 wide-field fluorescence images with minimal photobleaching. With this image sequence that could only be obtained under cryo-arrest, an SRRF reconstruction (*7*) of the ER of the same arrested cells became possible (Fig. 1E and fig. S2B).

### Cryo-arresting dynamic molecular patterns

**Fig. 2.**
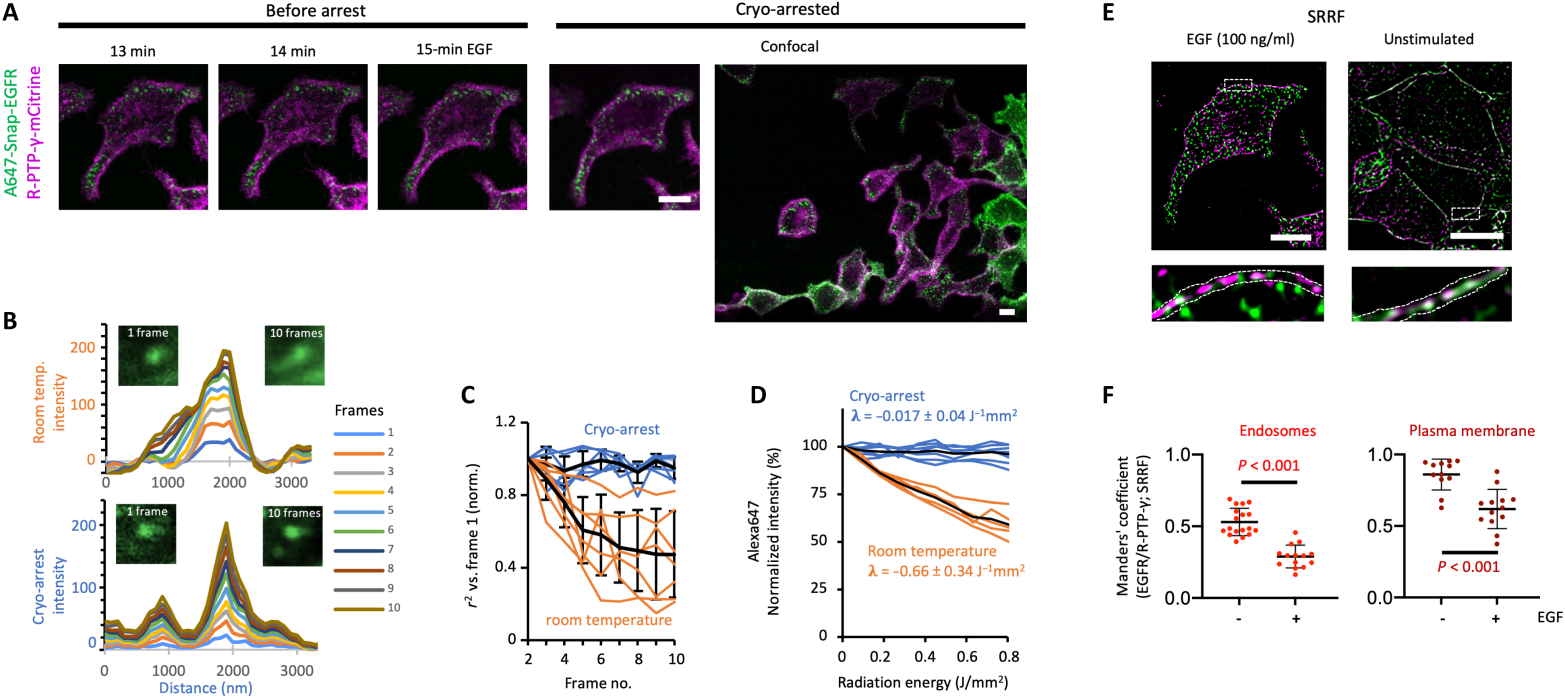
Cryo-arrest of a two-component molecular signaling system. (**A**) Dual-color CLSM images of R-PTP-γ-mCitrine (magenta) and Alexa647-Snap-EGFR (green) in MCF7 cells during EGF (100 ng/ml) stimulation before (at indicated time, 3.75-s scan time) and during cryo-arrest (10 frames, 37 s). Right: CLSM of a larger area during cryo-arrest. (**B**) Motional blur measured by CLSM of Alexa647-Snap-EGFR–labeled endosomes at room temperature (top) and under cryo-arrest (bottom). Left images: individual frame (2.5 × 2.5 μm); right image: sum of 10 frames. Graphs: corresponding cumulative background corrected line profiles color coded by frame number. (**C**) Motional blur measured by pattern similarity between frames. Lines: coefficient of determination (*r*^2^) of individual regions containing endosomes at room temperature (orange) and under cryo-arrest (blue); mean ± SD (black; *n* = 6). (**D**) Alexa647 photobleaching at room temperature (orange; *n* = 5) and under cryo-arrest (blue; *n* = 7) during 10 confocal scans. λ: photobleaching rates (mean ± SD). (**E**) Dual-color SRRF reconstruction from 100 wide-field frames of cryo-arrested cell depicted in (A) (left) and a cryo-arrested unstimulated cell (right). Bottom: magnifications of boxed areas; dotted lines: plasma membrane area. (**F**) Manders’ colocalization coefficients for Alexa647-Snap-EGFR/R-PTP-γ-mCitrine from SRRF reconstructions under cryo-arrest in endosomes (left) or plasma membrane area (right) for unstimulated (−EGF; *n* = 18) and 15-min EGF-stimulated cells (+EGF; *n* = 15); *P*: Student’s *t* test. Scale bars: 10 μm.

We next assessed how ultrarapid cryo-arrest could improve the investigation of a molecular signaling system whose spatiotemporal organization is highly dynamic. For this, we focused on the proto-oncoprotein EGFR (epidermal growth factor receptor) and the functionally related tumor suppressor R-PTP-γ (receptor-like protein tyrosine phosphatase γ) (*4*, *35*). We mounted MCF7 cells that coexpressed the fluorescent fusion constructs of EGFR (Alexa647-SNAP-EGFR) and R-PTP-γ fused to mCitrine (R-PTP-γ-mCitrine) on the cryo-microscope. The vesicular dynamics of both proteins at room temperature could be followed by CLSM upon stimulation with epidermal growth factor (EGF; 100 ng/ml), after which cryo-arrest allowed for precise capture of the spatial patterns of both proteins in relation to one another (Fig. 2A and fig. S3A). High-resolution colocalization analysis of the same arrested cells using SRRF reconstructions of 100 wide-field fluorescence images from each channel became possible because of the absence of motional blur (Fig. 2, B and C, and fig. S3, B and C) and enhanced photostability of the fluorescent markers (Figs. 1D and 2D). By comparing this to cryo-arrested unstimulated cells, the previously unknown co-organization of EGFR and R-PTP-γ into nanoscale clusters along the plasma membrane and in endosomes as well as their segregation upon EGF stimulus became apparent (Fig. 2, E and F, and fig. S4). This dynamic nanoscale organization between EGFR and R-PTP-γ thereby represents the resolution of a missing link of how growth factor receptor systems switch from a dynamically silenced to a transient active signaling state (*4*).

### Coordinate targeted cryo-nanoscopy of molecular patterns

**Fig. 3.**
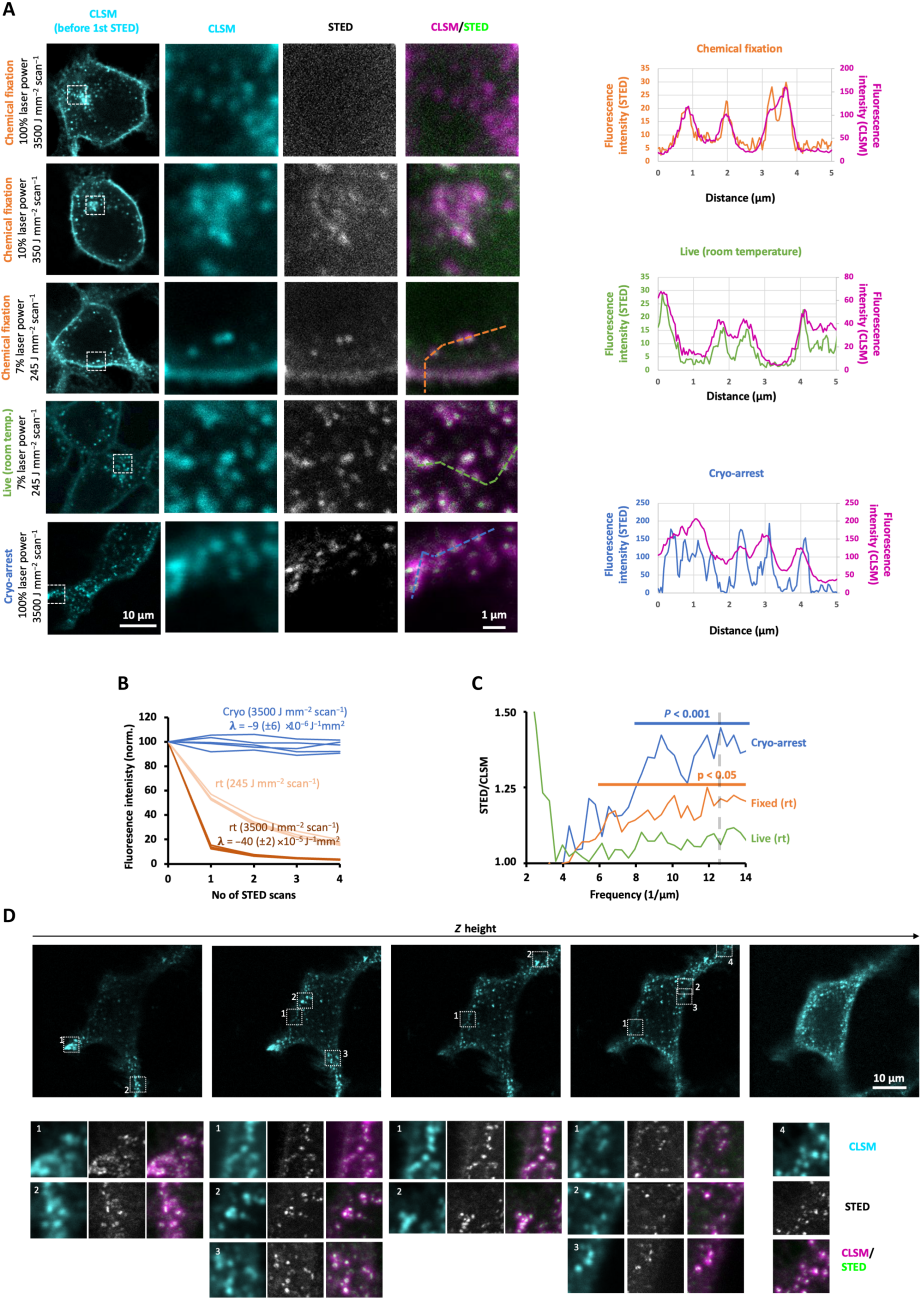
STED nanoscopy of a dynamic receptor system under cryo-arrest. (**A**) CLSM and STED nanoscopy of Alexa647-Snap-EGFR in chemically fixed (rows 1 to 3), live at room temperature (fourth row), and cryo-arrested (bottom row) MCF7 cells. CLSM of cells (first column; pixel size: 100 nm); detailed (pixel size: 40 nm) CLSM (second column) and STED (third column) scan of boxed area with indicated laser irradiation intensities. Fourth column: overlay, CLSM (magenta), and STED (green). Live: single scan; cryo-arrest: sum of 10 scans. Graphs: intensity profiles along the dotted lines depicted in the CLSM/STED overlay images (STED: color-coded to lines; CLSM: magenta). (**B**) Alexa647 photobleaching during STED scans at room temperature (rt) or under cryo-arrest (cryo) at the indicated depletion laser powers. λ: decay rates determined by exponential fits (mean ± SD, *N* = 5). (**C**) STED spatial frequency spectrum normalized by corresponding CLSM spectrum for different regions containing vesicles (cryo: *n* = 11; chemical fixation: *n* = 8; rt. live: *n* = 6); Horizontal lines: frequency range where STED information content is significantly higher than CLSM (Student’s *t* test); vertical dashed line: digital resolution. (**D**) Top row: 3D-CLSM of a cryo-arrested MCF7 cell. Lower rows: detailed high-resolution scans (5 × 5 μm; pixel size: 40 nm) of the indicated regions in the top row: CLSM (cyan), STED (gray), and merge (magenta and green).

STED nanoscopy constitutes a special case among superresolution techniques, because fluorescence intensity can be directly related to the density of fluorescence markers, yielding quantitative molecular patterns. However, in this laser scanning nanoscopy, resolution beyond the diffraction limit scales with the intensity of a second doughnut-shaped laser beam for stimulated emission of excited fluorophores (*9*). The consequent high demands to photostability, fluorescence quantum yield, and density of fluorescent labels severely hamper STED imaging of fluorescently tagged sparse molecules in live cells because of intense photobleaching at positive temperatures. Only a low-depletion laser power (<42 mW/μm^2^) preserved enough fluorescent markers to obtain a noisy STED image of Alexa647-Snap-EGFR in MCF7 cells at room temperature (Fig. 3, A and B). However, because of the low intensity of the depletion beam, there was no significant gain in resolution beyond the diffraction limit in comparison to regular CLSM (Fig. 3, A and C). Chemical fixation of the cells alleviated motional blur but only slightly improved resolution because of the limited amount of collected photons from five consecutive STED frames before completely bleaching the sample (Fig. 3, A and C). Yet, after ultrarapid cryo-arrest of living cells, the ~45-fold decreased bleaching rate (Fig. 3B) enabled a 10-fold increase in both the depletion laser power (420 mW/μm^2^) and exposure time (radiation energy density: 35 mJ/μm^2^), resulting in sharply resolved endosomal structures beyond the diffraction limit containing Alexa647-Snap-EGFR (Fig. 3, A and D). Resolution was significantly increased at scales <125 nm down to the digital (pixel-limited) resolution of 80 nm (Fig. 3C). No ice crystals were detected at this resolution as fluorescence-free areas in the cytoplasm of these cells (fig. S5). This was also the case after repeated STED scanning with maximal depletion laser intensity, after which the structure and configuration of the endocytic structures were preserved. This shows that the high-intensity STED illumination did not warm the sample enough to create ice crystals. Furthermore, because of the absence of motional blur and strongly reduced photobleaching, it was also possible to scan a whole cell in three-dimensions (3D) by CLSM and subsequently record superresolution STED images of Alexa647-Snap-EGFR in different regions at different *z* levels (Fig. 3D). This enabled the identification of vesicle clusters beyond the diffraction limit that could not be resolved at room temperature. Here, the total acquisition time was >15 min, amounting to an average irradiation energy density of >20 mJ/μm^2^ over the whole cell area, largely exceeding the lethal light dose of ~1 mJ/μm^2^ for mammalian cell lines at positive temperatures (*17*).

### Resolving reaction states by microspectroscopic imaging under cryo-arrest

**Fig. 4.**
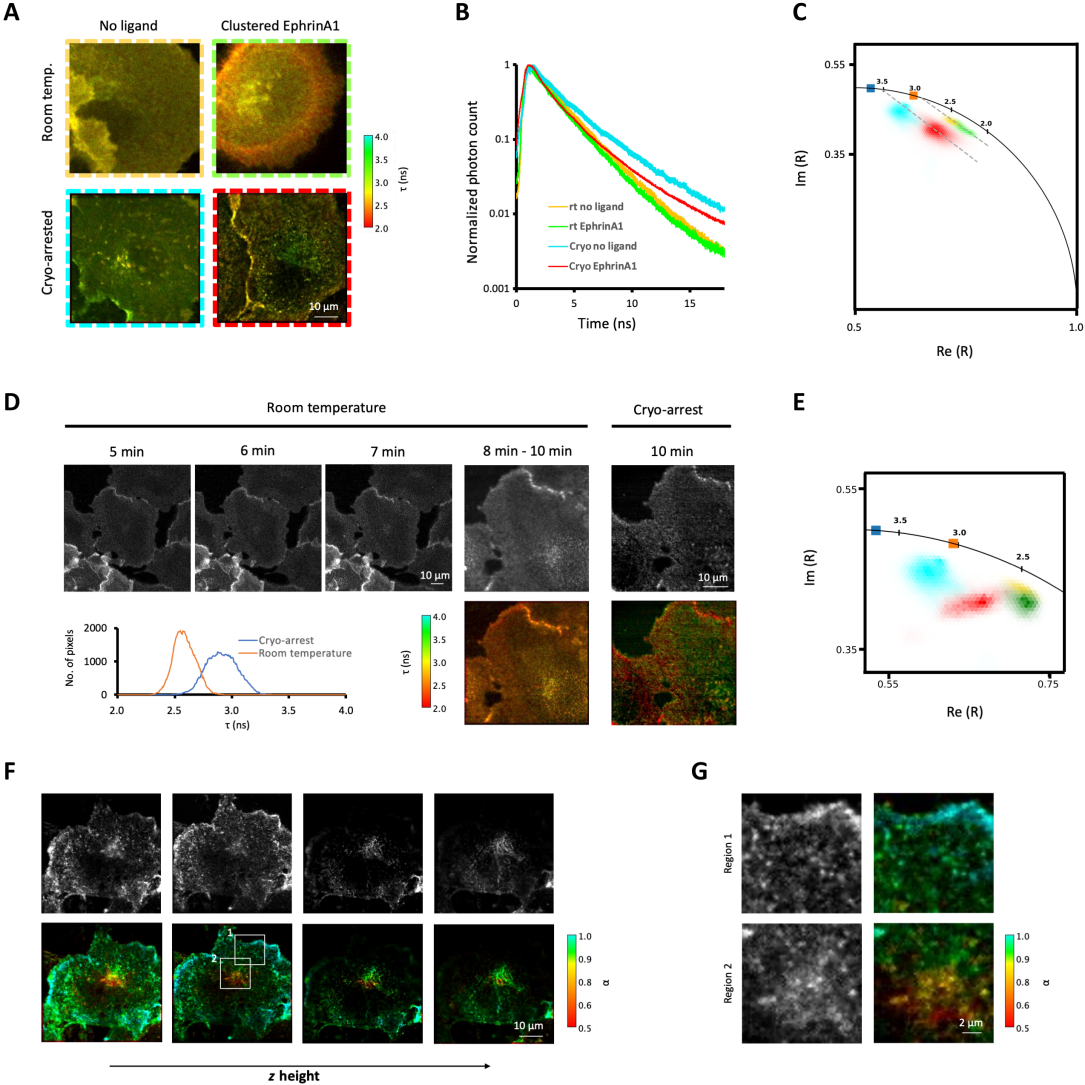
Resolution of spatially distributed reaction states under cryo-arrest. (**A**) Representative intensity-weighted average fluorescence lifetime (τ) images obtained by confocal FLIM of Cos7 cells stably expressing LIFEA2; without (left column) and with clustered EphrinA1 stimulation (right) at room temperature (rt; top) or under cryo-arrest (cryo; bottom). Color bar: τ (ns). (**B**) Normalized cumulative fluorescence decay histograms of LIFEA2 at room temperature and cryo-arrest, with (room temperature: green, *N* = 6, *n* = 9, cryo-arrest: red, *N* = 7, *n* = 16) and without (room temperature: yellow, *N* = 6, *n* = 10, cryo: cyan, *N* = 2, *n* = 11) clustered EphrinA1 stimulation. (**C**) Imaginary (Im) and real part (Re) of phasors (R) derived from the first harmonic of fluorescence decay data in (B); color-coded as in (B); squares: mCitrine only (blue: cryo-arrest; orange: room temperature; fig. S5, C and D); dashed lines: linear fit of phasors. (**D**) Confocal FLIM of a LIFEA2-expressing Cos7 cell after indicated times of clustered EphrinA1 stimulation before and during ultrarapid cryo-arrest. Top panels: fluorescence photon count; lower panels: τ (ns); graph: corresponding τ histogram at room temperature (orange) and cryo-arrest (blue). (**E**) Corresponding phasors of data in (D) (red: cryo-arrest; green: room temperature), with cumulative phasors from unstimulated cells in (C). (**F**) Fluorescence photon count (top panels) and fluorescence intensity–weighted fraction of the active conformation of LIFEA2 (bottom) at different *z* heights (rows) obtained by confocal FLIM upon cryo-arrest after 14-min stimulation with clustered EphrinA1. Color bar: fraction of LIFEA2 in active conformation (α). (**G**) Magnified regions of the boxed areas in (F).

We next assessed microspectroscopic imaging of molecular reactions in cells after ultrarapid cryo-arrest. Fluorescence lifetime imaging microscopy (FLIM) adds the spectroscopic dimension of nanosecond fluorescence decay kinetics to fluorescence microscopy (*12*, *36*). Molecular proximity–induced excited-state reactions between fluorophores [e.g., fluorescence resonance energy transfer (FRET)] can thereby be imaged to spatially resolve macromolecular reactions, such as interactions and conformational changes (*13*, *37*, *38*). The genetically encoded biosensor LIFEA2 (Linker optimized Intramolecular-FRET sensor for EphA2) reports on the molecular conformation and thereby kinase activity of the cell guidance ephrin receptor type A2 (EphA2) via FRET between the fluorescent proteins mCitrine and mCherry (*38*). At room temperature, receptor activation by the ligand clustered Ephrin A1 could be observed at the plasma membrane of Cos7 cells by a small (~0.2 ns) drop in average fluorescence lifetime of the FRET donor mCitrine in LIFEA2 (Fig. 4A and fig. S6, A and B) (*24*, *39*). Yet, motional blur and poor separation of the fluorescence decay profiles and derived phasors (Fig. 4, B and C) (*40*) severely limited the spatial and fluorescence lifetime resolution on membrane structures in the cell (Fig. 4A and fig. S6A). At cryo-temperatures, however, the increased quantum yield of mCitrine (fig. S6, C and D) resulted in a largely improved separation of the decay profiles, average fluorescence lifetimes (τ), and associated phasors of LIFEA2 in stimulated versus unstimulated cells (Fig. 4, A to C). Together with the alleviation of motional blur, clustered receptor activity could now be resolved in plasma membrane patches and endocytic structures (Fig. 4A and fig. S6E). Basal activity in the absence of exogenous stimulation became also apparent, likely caused by endogenous ephrin on neighboring cells. This contrast improvement in molecular activity imaging became especially evident when the same Ephrin A1–stimulated cell was compared before and during cryo-arrest (Fig. 4, D and E).

Under cryo-arrest, the large separation of the cumulative phasors (Fig. 4C) made it possible to derive the spatially invariant fluorescence lifetimes of the active (τ = 0.9 ± 0.2 ns) and inactive (τ = 3.5 ± 0.1 ns) conformations by global analysis (*38*) as well as the fraction of LIFEA2 with nonabsorbing mCherry acceptor (0.2 ± 0.05). Using these spatially invariant parameters, the spatially variant molecular fraction of active LIFEA2 (α) could be calculated from the phasor in each voxel of an image (*41*). Cryo-arrest thereby enabled the acquisition (time: >10 min) and reconstruction of a complete 3D molecular activity pattern of LIFEA2 in a stimulated Cos7 cell (Fig. 4F). The gain in spatial and spectroscopic resolution at cryo-temperatures thereby allowed to distinguish the activation state of a receptor tyrosine kinase (RTK) at the level of individual endocytic vesicles and receptor clusters (Fig. 4G). This resolution enabled the observation that EphA2 receptor clusters that emerge from the plasma membrane continue to signal from peripheral endocytic vesicles such as early endosomes, with only a gradual inactivation due to the phosphatase activity of ER-associated PTPs (*4*). However, the abrupt shutdown of EphA2 activity in individual endocytic vesicles that reach a defined perinuclear vesicular compartment could now also be resolved. Such a high-resolution, complete activity pattern obtained under cryo-arrest thereby sheds light on how receptor trafficking through spatially organized PTPs controls the temporal response of RTKs.

### Surpassing a fundamental resolution barrier by cryo-arrest

To distinguish whether the gain in resolution and localization precision under cryo-arrest are caused by changing fundamental physical parameters or can be surpassed by overcoming photon detection limitations, we investigated the theoretical limits of the interrelation between diffusion and photophysics that causes motional blur. In the absence of motion, the localization precision of a single emitter is defined as the square root of the variance (*v*) of its detected position, which can be calculated as (*42*)

$$v=\frac{\sigma_{\text{PSF}}^{2}+a^{2}/12}{N}+\frac{16\pi \;{\left(\sigma_{\text{PSF}}^{2}+a^{2}/12\right)}^{2}(b_{m}+b_{v}^{2})}{3a^{2}N^{2}}$$ (1)

where *a* is the pixel size, *b*_m_ and *b*_v_ are the mean and variance of the background photons, and σ_PSF_ is the SD of the Gaussian point spread function (PSF). In a specific experimental setup, *a*, *b*_m_, *b*_v_, and σ_PSF_ are fixed parameters. Thus, the number of collected fluorescence photons (*N*) has to be increased to improve localization precision. Because of the maximum in attainable photon flux that is independent of excitation energy (*16*), increasing the number of collected photons results in a longer acquisition time. A nonstationary particle changes its position during this time, which deteriorates the localization precision. The slowest movement of a structure is Brownian motion, which results, on average, in a Gaussian distribution of particles whose variance is the mean square displacement (MSD)

MSD=4DΔt (3)

where *D* is the diffusion coefficient and Δ*t* is the elapsed time. *D* for a structure of a given hydrodynamic radius (*r*) in a medium of certain viscosity (η) and temperature (*T*) is defined by the Stokes-Einstein relationship

$$D=\frac{1}{6\pi \eta r}k_{B}T$$ (4)

with the Boltzmann constant *k*_B_. Because the variance of the convolution of multiple Gaussians is the sum of their individual variances, the variance of the effective PSF under motional blur ($\sigma_{\text{MB}}^{2}$) that takes the radius (*r*) of the Gaussian image of a particle in account can be calculated as

$$\sigma_{\text{MB}}^{2}=\sigma_{\text{PSF}}^{2}+\text{MSD}+r^{2}$$ (5)

**Fig. 5.**
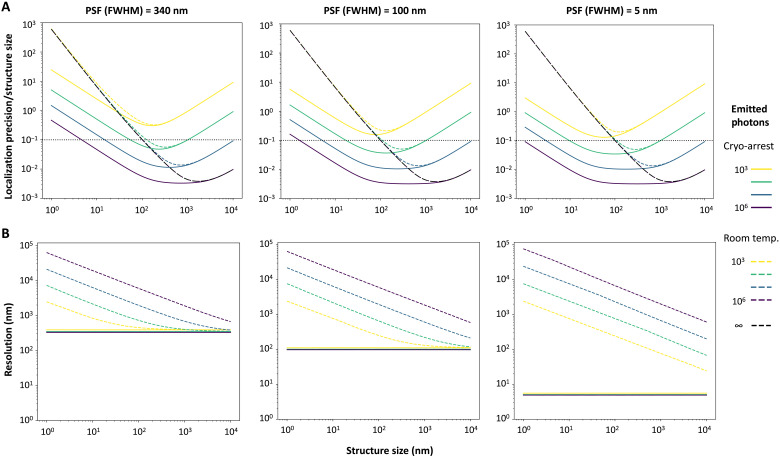
Calculated localization precisions and resolutions at room temperature and under cryo-arrest. Calculated localization precision relative to the actual structure size (**A**; horizontal black dotted line: localization precision with 10% of actual structure size) and resolution (**B**) plotted against the structure size for different amounts of emitted photons (color code; right) at room temperature with simulated Brownian motion (dashed lines) or under cryo-arrest (continuous lines) for different PSF defined by their full width at half maximum (FWHM). For the calculations of localization precision and resolution, a typical 10% photon collection efficiency of the microscopy detection system was considered.

By replacing $\sigma_{\text{PSF}}^{2}$ by $\sigma_{\text{MB}}^{2}$ in Eq. 1, the relative localization precision in relation to particle size can be calculated (Fig. 5). Calculations were performed for three different $\sigma_{\text{PSF}}^{2}$, ranging from high-NA air objectives [full width at half maximum (FWHM): ~340 nm] to very small PSFs that could be obtained by coordinate-targeted nanoscopy such as STED. Taking detector noise, a realistic photon collection efficiency (*16*), and a photon emission rate of 2 μs^−1^ that prevents fluorophore reexcitation and minimizes bleaching (*43*, *44*) into account, this showed a fundamental limit to localization precision that depends on particle size at positive temperatures caused by Brownian motion (black dashed lines in Fig. 5A). This limit is independent of $\sigma_{\text{PSF}}^{2}$ of the microscope or the number of emitted photons. Resolution, defined as the Gaussians images of two objects being separated on the basis of the Abbe criterion, was determined using a Monte Carlo simulation of photon emission from a bimodal Gaussian distribution defined each by $\sigma_{\text{MB}}^{2}$ and separated by distance *d* as a function of photon number. These simulations showed that resolution is also fundamentally limited by Brownian motion at positive temperatures. Here, motional blur outweighs the number of collected photons, and resolution cannot be further improved by prolonging acquisition times (Fig. 5B). For coordinate-targeted nanoscopy, which can achieve very high resolutions because of engineering of the PSF, a decrease in resolution is evident over the whole range of (sub)cellular structures (Fig. 5B, right graph).

Because the viscosity of water increases by at least 15 orders of magnitude between 37° and −196°C (*44*), diffusion becomes negligible. Because of this, the fundamental limitations on resolution and localization precision can be surpassed under cryo-arrest. For example, for a 100-nm spherical structure that emits 10^5^ photons at an emission rate of 2 μs^−1^, resolution is ~20-fold (with a PSF of FWHM = 100 nm), and localization precision ~10-fold improved by cryo-arrest (10-nm structure: 60- and 100-fold, respectively) (Fig. 5). Because of the strongly reduced bleaching in the absence of molecular motion under cryo-arrest, the resolution and localization precision can be even further improved by collecting more photons from the immobile structures.

## DISCUSSION

We presented ultrarapid cryo-fixation that allows the arrest of native molecular patterns during microscopic measurements and their subsequent high-resolution observation in living mammalian cells. Other cryo-fixation approaches were developed for electron microscopy and adapted to fluorescence microscopy, such as plunge freezing or high-pressure freezing. These approaches allowed breakthrough measurements in structural biology as well as fluorescence microscopy and spectroscopy (*26*, *45*, *46*). Yet, they do not allow connection between the measurement of collective molecular dynamics and the high-resolution measurement of molecular patterns under cryo-arrest. Furthermore, it cannot be verified whether cell structures and patterns were preserved by the arrest. Previous approaches to rapidly cryo-arrest biological samples directly on a microscope were used to cryo-fix microorganisms during light microscopy for subsequent electron microscopy analysis (*47*). However, the cooling was not fast enough to avoid ice crystal formation in the medium despite the application of cryoprotectants because of the presence of a heater and insulator between the coolant and the sample (*47*). Cryoprotectants affect the sensitive physiological balance of mammalian cells (*24*, *48*, *49*), which are also much more susceptible to ice crystal damage because of their higher water content than microorganisms such as yeast or *Caenorhabditis elegans* (*50*). In the approach presented here, the mounting of the sample directly on a diamond with superior thermal conductivity allowed for high enough cooling speeds to preserve mammalian cells even without application of cryoprotectants. We consequently did not observe ice crystals in a dilute aqueous medium and also not beyond the diffraction limit within cells by STED microscopy. Also, cellular structures imaged beyond the diffraction limit by STED or SRRF showed no distortions. We can thus conclude that at all resolutions that we could access, there was no evidence of ice crystals. The same remained true after repeated scanning with the STED laser, showing that this high-intensity but very local (<1 μm^2^) and short-duration (<10 ms) irradiation was not warming the sample enough to create detectable ice crystals. This is likely due to efficient heat dissipation from the small illuminated volume in the sample mounted on a diamond of superior thermal conductivity. Furthermore, the distribution of the molecular conformations of the LIFEA2 sensor as measured by FRET-FLIM was preserved, indicating that protein structure remains intact after cryo-arrest. Thus, the ultrahigh speed of cryo-fixation faithfully arrests a temporal state of dynamic molecular patterns in cells that can be observed at multiple resolutions.

Our results also show that motional blur and photoreactivity set fundamental limits on localization precision and spatial resolution, which can be surpassed by cryo-arrest. It is of note that methodological constraints that affect photon acquisition times, such as photoswitching, laser scanning or sequential acquisition of multiple channels, and active motion of particles, will further decrease resolution at positive degrees Celsius temperatures. The increased quantum yield at −196°C additionally increased spectroscopic resolution in FLIM, which made the acquisition of complete molecular reaction patterns possible. Furthermore, the strongly reduced photochemistry allowed attaining superresolution in STED nanoscopy of fluorescently tagged sparse molecules by enabling high-intensity irradiation by the depletion beam that defines resolution. The reduced photochemistry also enables multimodal microscopy to observe patterns at multiple scales as demonstrated for the CLSM/SRRF and CLSM/STED imaging of the EGFR/R-PTP-γ system. This revealed nanoscopic features of a dynamical signaling system that were obscured during live cell imaging.

Because of the accessible optical configuration with the sample on a standard microscopy cover slide, this approach is compatible with all existing inverted, epifluorescence microscopes and thus with a vast majority of nano- and microspectroscopic fluorescence imaging techniques. The higher photostability at cryo-temperatures more than outweighs the collection efficiency limitations of air objectives for localization of individual molecules (*45*, *51*). However, recent technical developments like cryo-immersion (*52*) or solid-immersion lenses (*53*) with NAs >1 could be combined with ultrarapid cryo-arrest on the microscope to even further improve spectroscopic and spatial resolutions of native molecular patterns in cells.

## MATERIALS AND METHODS

### Design and operation of the ultrarapid cryo-fixation device

The main parts of the cooling device are a vessel for the pressurized mixture of N_2_ and helium and the heat exchanger unit (see Fig. 1A). The pressure vessel was made from stainless steel and was sealed with copper gaskets at the junctions. It is cooled from the outside by liquid N_2_, which is filled into a vessel made from steel sheet and insulated by Styrodur. It contained a pneumatically controlled custom-built piston, which functions as a valve (Key Science Engineering). The piston was sealed with spring-loaded PTFE (polytetrafluoroethylene) sealings at the shaft (Gesellschaft für Dichtungstechnik mbH). The tip of the piston was made from high-density polyethylene (HDPE) to form a sealing against the stainless steel of the vessel when it is closed and at the same time be resistant to the pressure. When the piston is lifted, the pressurized fluid is released to the heat exchanger unit (Key Science Engineering; Fig. 1A). The connecting part was kept as short as feasible, and it was insulated from the inside by HDPE to minimize heating of the coolant from the metal parts. Within the heat exchanger unit, the cooling fluid was guided through a nozzle directly on the CVD diamond (Element Six Technologies Ltd., Aston; red arrow in Fig. 1A). Afterward, it can expand and is guided toward the exhaust (green arrows in Fig. 1A). Using a mixture of helium and N_2_ provided much better cooling performances than each of the two alone. When N_2_ alone is inserted into the vessel at 50 bars, it liquifies rapidly at −196°C, which leads to a pressure drop in the vessel and thus a slow release. Consequentially, cooling rates were much slower. Helium cannot liquify under these conditions, but the gas alone has a too low thermal capacity, leading to an incomplete and slow cryo-arrest. Thus, N_2_ was loaded into the device by first filling it with nitrogen from an in-house supply (approximately 7 to 8 bars), which was guided through a copper spiral immersed in liquid nitrogen, for 1 min. Afterward, the pressure was increased by helium from a pressurized bottle (200 bars, Westfalen AG) to 50 bars using a reducer valve. All valves were controlled via a graphical user interface, which was created using the LabVIEW software (National Instruments). A check valve suitable for cryogenic use (CGS250SS, Flüssiggas-Anlagen GmbH) was installed between the pressure vessel and the copper spiral for the nitrogen supply to prevent the high pressure from entering the copper spiral. The whole setup was mounted on a frame above the epifluorescence microscope in a way that it could be elevated for sample mounting using an electronically controlled lifting cylinder (1000 N, Drive-System Europe Ltd.).

Temperature before cooling was measured close to the sample by a thermocouple and was regulated by a PID (power integral derivative) controller (KT4; Panasonic Corporation, Kadoma, Japan), which controlled two 50-W heating bands (Friedr. Freek GmbH) that were mounted to the heat exchanger unit. Setting the temperature to 37°C and measuring inside of the sample every 0.2 s over the time course of 60 min using a 50-μm constantan-copper thermocouple resulted in a measurement of 36.3° ± 0.5°C (mean ± SD) within the sample. To keep the temperature constant at −196°C after the rapid cooling, N_2_ from the in-house supply (approximately 8 bars), which was guided through a copper spiral immersed in liquid nitrogen (Fig. 1A), was used. The check valve between the copper coil and the pressure vessel opens automatically, when the pressure in the vessel drops below the pressure in the copper coil. Thereby, liquid nitrogen flows through the vessel on the diamond and keeps it at temperatures close to −196°C after a transition phase, during which the glass transition temperature (approximately −136°C) of water is not reached (Fig. 1B).

Around the objective, a transparent flexible foil was mounted by a custom 3D-printed connector to the stage. The room inside of this foil was filled with dry helium at room temperature at a constant low flow throughout the experiment. The temperature of the objective was regulated by an objective heater (Bioptechs Inc.). The objective heater was always set to 25°C. After 30 to 60 min of imaging under cryo-arrest, the measured temperature of the objective was usually between 7° and 10°C.

### Calculations of resolution and localization precision

We calculated the resolution and localization precision by considering molecular motion by Brownian diffusion, the fluorophore’s quantum yield and photostability, and the photon detection efficiency of the imaging system. Motion was modeled as diffusion of molecules through low Reynolds number liquid, and therefore, the diffusion coefficient ***D*** for an object was obtained as a function of temperature (***T***) and hydrodynamic radius (***r***) through the Stokes-Einstein equation (Eq. 4), where **η** is the viscosity of the liquid and ***k***_**B**_ is the Boltzmann constant. The viscosity of water at 37°C is 0.6922 mPas. Upon cryo-arrest, objects were considered to be static because viscosity is increased by >15 orders of magnitude (*54*). The photophysical properties of fluorescent proteins (quantum yield and photostability) were obtained from literature, and their change upon cryo-arrest was obtained from the data presented here [QY_CA_/QY_RT_ = 1.2, (Emitted photons)_CA_/(Emitted photons)_RT_ > 10].

Using a Gaussian approximation for the PSF of the microscope and using that the variance of the convolution of multiple Gaussians is the sum of their individual variances, the variance of the PSF that includes motional blur ($\sigma_{\text{MB}}^{2}$) can be calculated as

$$\sigma_{\text{MB}}^{2}=\sigma_{\text{PSF}}^{2}+v_{\text{Bm}}$$ (6)

where *v*_Bm_ is the variance that results from the Gaussian distribution of particles resulting from Brownian motion and equals the MSD of the diffusion process (Eq. 3). This $\sigma_{\text{MB}}^{2}$ would be appropriate for localizing a single point-like light source on a structure of a given size. In contrast, when the structure of interest is uniformly labeled with many fluorophores, the observed object has to be additionally convolved with the size of the object. Using a Gaussian approximation for the 2D image of the structure, the output variance is again the sum of the input variances. Thus, $\sigma_{\text{MB}}^{2}$ was calculated as in Eq. 5. Calculations were performed as a function of the number of photons emitted by the molecule at a rate of 2 μs^−1^ at room temperature. This value ensures that fluorophores are not reexcited when active and was found to be in accordance with other values reported in literature (*43*, *44*). In addition, it was considered that only 10% of the emitted photons arrived at the detector because of reduced collection and transmission efficiency.

The resolution as a function of the number of photons was calculated using a Monte Carlo simulation. Briefly, ***N*** photons were drawn from a distribution consisting of two Gaussians defined by $\sigma_{\text{MB}}^{2}$ and separated by a given distance ***d***. The outcome was binned (size ***a***) to simulate pixelation. A Gaussian mixture model was used to recover these Gaussians, and the contrast was defined as

$$C=1-\frac{I_{\text{min}}}{I_{\text{max}}}$$ (7)

where *I*_min_ corresponds to the number of counts in the valley between the two Gaussians, and *I*_max_ corresponds to the maximum height of the two peaks. The structures were considered to be resolved when *C* > 1.7%, following the Abbe criterion. This process was repeated 1000 times for each combination of $\sigma_{\text{MB}}^{2}$, *d*, and *N*. When the distance was larger than 3 $\sqrt{\sigma_{\text{MB}}^{2}\;}$, the structures were considered resolved. The resolution for a given $\sigma_{\text{MB}}^{2}$ and *N* was defined as the minimum *d* at which 95% of the realizations were resolved. The localization precision was calculated as a function of *N*, *a*, and the noise of the detector using Eq. 1 (*42*, *55*, *56*). The $\sigma_{\text{PSF}}^{2}$ was replaced by the $\sigma_{\text{MB}}^{2}$ to account for motional blur. Note that the size of the PSFs in Fig. 5 is described by the FWHM (= 2.355 σ) because this value matches closely the resolution given by the Abbe criterion.

### Measurement of cooling rates

The cooling performance was measured by a 50-μm constantan-copper thermocouple (Omega Engineering Inc.; response time: 3 ms) mounted in a microliter drop of aqueous buffer. Because of the thickness of the thermocouple, the total thickness of the sample was **~**100 μm, as inferred from the drop covering the whole area of the mounted cover slide. The thermocouple was connected via a USB data acquisition module (Omega Engineering Inc.) to a computer and readout with a frequency of 1000 Hz (digital resolution: 2 ms) using the LabVIEW software (National Instruments).

### Calculation of thermal diffusivity–limited cooling rates

To relate the measured cooling rates (fig. S1A) to thermal diffusivity–limited cooling rates in the aqueous sample, heat flux–limited cooling from one surface (i.e., cooling the aqueous solution from the diamond) was based on the equation for the rate of heat flow

$$\frac{\mathrm{dQ}}{\mathrm{dt}}=-\kappa A\frac{\Delta T}{L}$$ (8)

The area (*A*) is that of the cover slide (2 × 10^−5^ m^2^). The heat conductivity (κ) of water is 0.5562 Wm^−2^, and the initial temperature difference (Δ*T*) is approximately 233°C between a sample at 37°C and liquid nitrogen (−196°C). The thickness (*L*) was calculated from the pipetted volume covering the whole cover slide. The freezing speed of the sample containing the 50-μm thermocouple was calculated by assuming that the thermocouple is measuring at 50-μm depth (*L* = 50 μm) within the 100-μm-thick sample. For aqueous samples without thermocouple, we measured a thickness of 10 to 15 μm and assumed a typical *L* ~ 13 μm for adherent mammalian cell samples.

The heat flow Q can also be described as

Q=c*m*T (9)

with *c* being the heat capacity (water: 4 J cm^−3^ K^−1^) and *m* being the mass of the sample (0.25 mg).

Combined, this results in

$$\frac{\mathrm{dT}}{\mathrm{dt}}=-\kappa A\frac{\Delta T}{\text{cmL}}$$ (10)

The estimated temperature course for a sample of thickness *L* has been calculated using MATLAB starting from the initial temperature of 37°C

$$T=\Delta T[e^{-\kappa A/(\mathrm{cmL})}-1]+T_{0}$$ (11)

Despite neglecting heat transport along the thermocouple and the different thermal conductivity and capacity of the thermocouple and the aqueous solution, the resulting diffusivity-limited and measured temperature gradients were very similar (fig. S1, A and B).

### Generation of stable transgenic cell lines

A polyclonal stable cell line of MCF7 expressing TC-PTP fused to mCitrine (TC-PTP-mCitrine) was generated on the background of MCF7 cells, which have been modified by CRISPR gene editing to not express endogenous TC-PTP. TC-PTP-mCitrine (*35*) was first cloned into the PiggyBac vector (System Biosciences). Afterward, MCF7 cells were transfected with this construct and PiggyBac transposase (System Biosciences) in a 1:1 ratio. Transfections were performed 24 hours after seeding using FuGENE 6 (Roche Diagnostics) as transfection reagent. The transfection was performed according to the manufacturer’s protocol. After 1 day, transfected cells were selected by puromycin (2 μg/ml) for 30 hours. After one additional week in regular cell culture medium, the cells were sorted by fluorescence-activated cell sorting (FACS) for high TC-PTP-mCitrine expression.

A polyclonal Cos7 cell line stably expressing LIFEA2 (*38*) was generated by cloning LIFEA2 into the PiggyBac vector (System Biosciences). Afterward, MCF7 cells were transfected with this construct and PiggyBac transposase (System Biosciences) in a 1:1 ratio. Transfections were performed 24 hours after seeding using FuGENE 6 (Roche Diagnostics) as transfection reagent. The transfection was performed according to the manufacturer’s protocol. After 1 day, transfected cells were selected by puromycin (3 μg/ml) for 14 days.

To generate a polyclonal stable MCF7 cell line expressing EGFR with an extracellular SNAP-tag (SNAP-EGFR) (*57*) and R-PTP-γ fused to mCitrine (R-PTP-γ-mCitrine) (*35*), both constructs were first cloned into the PiggyBac vector (System Biosciences). Afterward, MCF7 cells were transfected with the two constructs and PiggyBac transposase (System Biosciences) in a 1:1:2 ratio. Transfections were performed 24 hours after seeding using FuGENE 6 (Roche Diagnostics) as transfection reagent. The transfection was performed according to the manufacturer’s protocol. After 1 day, transfected cells were selected by puromycin (2 μg/ml) for 30 hours. After one additional week in regular cell culture medium, the cells were sorted by FACS for high R-PTP-γ-mCitrine expression. Labeling of Snap-EGFR was done by incubating the cells with 0.5 μM Snap-Surface Alexa647 (New England Biolabs GmbH) for at least 60 min. Because of the constant recycling of EGFR, this resulted in labeling of EGFR at the plasma membrane and in endocytic compartments. The cells were labeled with Snap-Surface Alexa647, and it was microscopically confirmed that >99% of these cells also express SNAP-EGFR.

### Cell culture, transfection, and preparation

HeLa [American Type Culture Collection (ATCC) no. CCL-185], Cos7 (ATCC no. CRL-1651), and MCF7 (ATCC no. HTB-22) cells were obtained from ATCC. The cells were authenticated by short tandem repeat analysis and did not contain DNA sequences from mouse, rat, or hamster (Leibniz Institute DSMZ). The cells were regularly tested for mycoplasma infection using the MycoAlert Mycoplasma Detection Kit (Lonza). They were maintained in Dulbecco’s modified Eagle’s medium (DMEM) supplemented with 10% fetal bovine serum, 200 mM l-glutamine, and 1% nonessential amino acids and cultured at 37°C with 95% air and 5% CO_2_.

For ultrarapid cryo-arrest experiments, cells were either seeded on 4-mm cover slides in 3.5-cm plastic petri dishes at a concentration of 1 × 10^5^ to 2 × 10^5^ cells per well or the 4-mm cover slides were attached to chambers from biocompatible silicone (4-well micro-Inserts; ibidi GmbH), which were filled with 20 μl of cell culture medium of 4 × 10^4^ cells/ml. The latter approach had the advantage that the bottom side of the cover slides remained free of medium. Cells were seeded at least 1 day before the experiment. Excess liquid was removed before the experiment, and the cover slides were placed on a custom 3D-printed holder to facilitate their mounting by adhesion of the remaining aqueous solution directly on the diamond heat exchanger. Experiments at 37°C were performed in glass-bottom cell culture dishes (MatTek Corporation) with 1 × 10^5^ to 4 × 10^5^ cells per dish.

Transient transfections of HeLa cells with EGFP (GFP-N1 vector, Clontech Laboratories Inc.), have been done 6 to 24 hours after seeding of the cells using 1 μg of DNA per well and FuGENE 6 (Roche Diagnostics) as transfection reagent. The transfection was performed according to the manufacturer’s protocol.

Chemical fixation for STED imaging was done by incubating the cells in 2% glutaraldehyde in 4% formaldehyde in phosphate-buffered saline at 37°C for 20 min. This fixes cells faster and with less artifacts than other aldehyde fixations (*21*).

### Fluorescence microscopy

Cryo-fluorescence microscopy and corresponding control experiments were performed on a confocal laser scanning STED microscope (Expert Line, Abberior Instruments) equipped with 488-, 561-, and 640-nm excitation lasers; STED lasers at 595 nm (nominal 1 W); and 775-nm (1.25 W) wavelength and FLIM acquisition hardware and software integration. The system was additionally equipped with a MT20 illumination system (Olympus Deutschland GmbH) and an Orca-R2 camera (Hamamatsu Photonics) for wide-field imaging.

Wide-field fluorescence imaging of HeLa cells expressing EGFP and solutions of rhodamine 6G and donor-only controls for FLIM were done with a 40× 0.6-NA objective (LucPlanFLN; Olympus Deutschland GmbH). All other images have been acquired with a 40× 0.95-NA objective (UPlanApo; Olympus Deutschland GmbH).

Confocal imaging including FLIM measurements has been performed by 485-nm laser excitation (mCitrine) or 640-nm laser excitation (Alexa647; measured irradiation power at the sample plane for photobleaching: 0.2 mW) and pixel length of 100 or 200 nm for confocal FLIM. Wide-field fluorescence imaging has been done with filter sets for EGFP and mCitrine (excitation: 470/40 nm; dichroic mirror: 495 nm; emission: 520/35 nm; measured power at the sample plane: 10 mW) or for Alexa647 (excitation: 620/60 nm; dichroic mirror: 640 nm; emission: 700/75 nm) and pixel length of 163 nm. Sequential wide-field fluorescence images for SRRF reconstructions have been acquired with a frame rate of 1 frame/s. This comparably low frame rate allowed successful reconstruction of the superresolution images from the reduced fluorescence fluctuation dynamics under cryo-conditions.

STED imaging was done with 640-nm laser excitation and the 775-nm laser for STED. Samples have been scanned with a pixel length of 40 nm and a dwell time of 15 μs. Individual frames have been aligned by rigid body registration algorithm using the open-source program Fiji (*58*) to compensate for sample drift. To achieve the optimal doughnut-shaped depletion beam under cryo-conditions, spherical aberrations had to be corrected by the correction collar of the objective.

### Image analysis

General image processing including quantification of fluorescence intensities, extracting line profiles, and calculation of Manders’ coefficients was done using the open-source program Fiji (*58*). Bleaching rates were estimated by fitting fluorescence bleaching sequences with a monoexponential function plus an offset using Microsoft Excel (Microsoft Corporation, Redmond, WA, USA). SRRF reconstruction was done using the NanoJ plugin to Fiji (*59*). Coefficients of determination were calculated using the pearsonr function implemented in SciPy (version 1.5.2) (*60*).

### Analysis of FLIM datasets

Each photon-counting dataset was registered to compensate for possible stage drift by applying a rigid translation-only transformation. After calculating a photon-counting image stack, the shifts in *x* and *y* directions of each frame in relation to the first were found using a discrete Fourier transform–based algorithm (*61*) implemented in Python (imreg_dft 2.0). These values were then rounded to the nearest integer. A new, registered photon-counting dataset was assembled by shifting each photon in each frame by the number of pixels previously determined. Photons that fell outside of the image defined by the first frame after registration were dropped.

Registered photon-counting datasets were corrected for background and instrument response function (IRF) directly from the data as previously described (*62*). Briefly, the background was estimated in two ways. First, all frames in the image stack were summed together to produce a photon-counting image. After denoising using a 9-pixel median filtered implemented in Python (SciPy 1.5.2), the position (*P*) of the minimum value was found. The total background was then calculated from the original image as the mean value around P pixel using a 21 pixel–to–51 pixel (depending on the image) window. The background was also estimated from the photon-counting histogram. Again, after median denoising (21-bin window), the background per bin was estimated as the mean value (51-bin window) in the original histogram around the minimum in the denoised one. This value was then extrapolated to the whole image by multiplying by the number of bins. Both background values were cross-checked for consistency. The IRF was estimated from the higher harmonics of the histogram.

The resulting corrected datasets were denoised using pawFLIM (*41*). Briefly, this is a weighted translational-invariant Haar wavelet transform denoising algorithm to phasor images, which results in significantly less bias and mean square error.

Measured average fluorescence lifetimes of the FRET donor mCitrine fused to TC-PTP or R-PTP-γ expressed in MCF7 cells increased from 3.04 ± 0.01 ns at room temperature to 3.71 ± 0.02 ns (mean ± SD) under cryo-arrest (*P* < 0.0001 using Student’s *t* test) but remained close to monoexponential (fig. S6, C and D). In measurements using LIFEA2, the clearly resolved nonexponential fluorescence decay profiles under cryo-arrest indicated mixtures of active and inactive conformations that exhibit a distinct average FRET efficiency and a fraction of LIFEA2 with nonabsorbing mCherry acceptors. Thus, the resulting denoised cumulative phasor plots were analyzed by global analysis with a three-component model derived as an extension of the two-component model previously described (*38*). These three spatially invariant states are (i) a fixed fraction of donor alone (sensor with nonabsorbing acceptor; fraction under cryo-arrest: 0.2 ± 0.05) and fluorescence lifetime (3.71 ± 0.02 ns), (ii) sensor in closed (active) conformation with fluorescence lifetime (0.9 ± 0.2 ns) and corresponding FRET efficiency (0.75 ± 0.25), and (iii) sensor in extended (inactive) conformation with fluorescence lifetime (3.5 ± 0.2 ns) and corresponding FRET efficiency (0.05 ± 0.01), each represented by a phasor. Therefore, each observed phasor is the sum of three phasors weighted by the corresponding normalized number of photons from each state. The complete phasor data of an image were therefore fitted with a linear combination of the three phasors and the corresponding photon fractions using a custom-made Python program based on lmfit 1.0.2. As previously described (*36*), the conversion from photon fractions to molecular fractions in each state was done by dividing each by the corresponding fluorescence lifetime (a measure of the quantum yield) and renormalizing the sum to 1. The fraction of active conformation was renormalized by dividing by (1 − fraction of donor alone) to account for the fraction of sensors with nonabsorbing acceptors.

## Notes

View/request a protocol for this paper from *Bio-protocol*.
