# Supplementary material for: Ultrarapid cryo-arrest of living cells on a microscope enables multiscale imaging of out-of-equilibrium molecular patterns
Source: Sci Adv. 2021 Dec 10;7(50):eabk0882. doi: 10.1126/sciadv.abk0882 (PMC8664253; doi:10.1126/sciadv.abk0882)
Supplement: Supplementary file 1 — Figs. S1 to S6 [file sciadv.abk0882_sm.pdf]

Science Advances
AAAS

Supplementary Materials for

Ultrarapid cryo-arrest of living cells on a microscope enables multiscale imaging of out-of-equilibrium molecular patterns

Jan Huebinger, Hernan Grecco, Martín E. Masip, Jens Christmann,
Günter R. Fuhr, Philippe I. H. Bastiaens*

*Corresponding author. Email: philippe.bastiaens@mpi-dortmund.mpg.de

Published 10 December 2021, *Sci. Adv.* **7**, eabk0882 (2021)
DOI: 10.1126/sciadv.abk0882

This PDF file includes:

Figs. S1 to S6

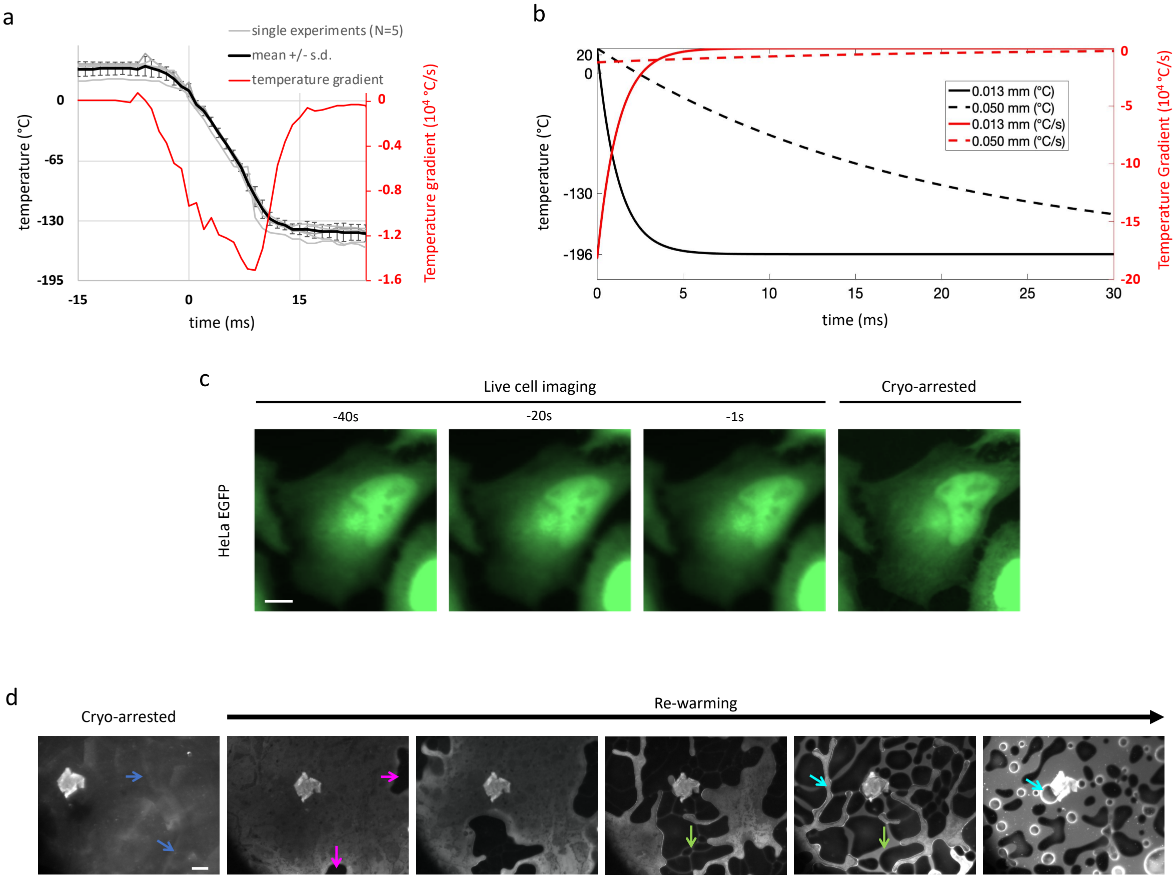

Fig. S1. Performance of ultra-rapid cryo-arrest

a) Measured cooling performance of the ultra-rapid cooling device in 100-µm thick aqueous samples containing a thermocouple (N=5) **b)** Calculated maximum conductive cooling in aqueous samples with thickness of 0.013 mm thickness (continuous lines) or 0.05 mm thickness (dashed lines) from 37 °C to -196 °C; black: temperature; red: temperature gradient **c)** Widefield epifluorescence microscopy of a HeLa cell transfected with EGFP at the indicated times before and under cryo-arrest at -196 °C (rightmost image). **d)** Leftmost image: Widefield fluorescence image of the fluorescent dye rhodamine 6G in cell culture medium after ultra-rapid cryo-arrest on the microscope. The sample was mostly homogeneous apart from a highly fluorescent structure, which was already present before the cryo-arrest. Blue arrows: expansion cracks. Images 2-6: Same sample during re-warming. Cracks disappeared completely, before ice crystals became visible by exclusion of the fluorescent dye (magenta arrows). Subsequently, the ice crystals first grew and melted at even higher temperatures into an expanding liquid phase between the ice crystals (exemplified by green arrows). Below parts of the liquid phase an accumulation of gas can be observed that later forms bubbles in the liquid phase (exemplified by cyan arrows). Scale bars: 10 µm

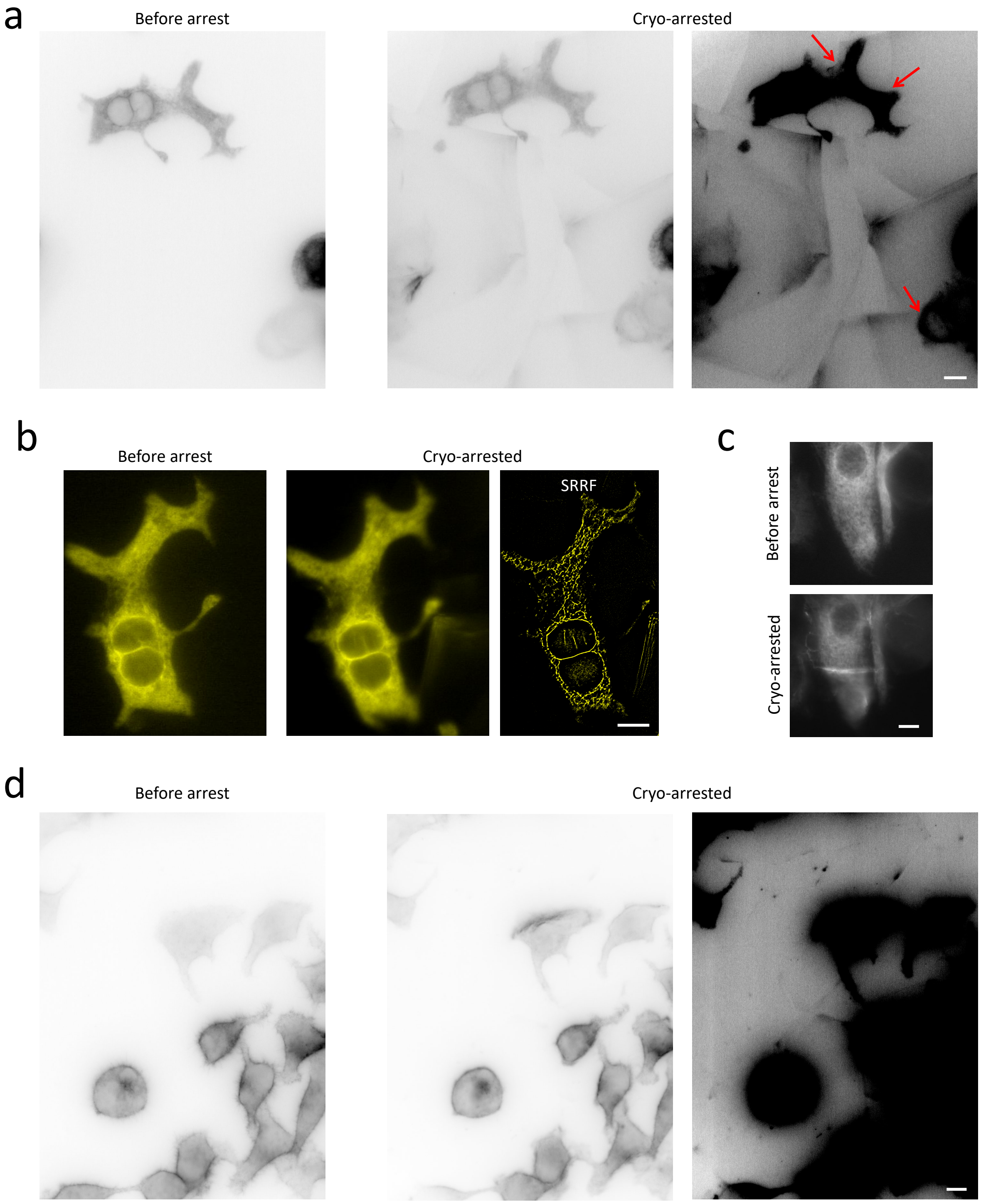

Fig. S2: Widefield microscopy before and under cryo-arrest

a-c) Widefield fluorescence microscopy of the fluorescent endoplasmic reticulum marked by TC-PTP-mCitrine in MCF7 cells. **a)** Micrographs before (left image) and during cryo-arrest (middle and right images; different contrast). Numerous cracks are detectable in the medium of this sample that reach the cells (red arrows). **b)** Magnification of the upper cell in (a) before and during cryo-arrest including a SRRF reconstruction from 100 images taken under cryo-arrest. **c)** Example of a cell showing damage by a crack traversing the cell. **d)** Widefield fluorescence microscopy of R-PTP-γ-mCitrine in MCF7 cells (left image) and during cryo-arrest (middle and right images; different contrast). Some cracks are detectable in the medium that sometimes reach a cell, but most cells appear intact. scale bars: 10 μm

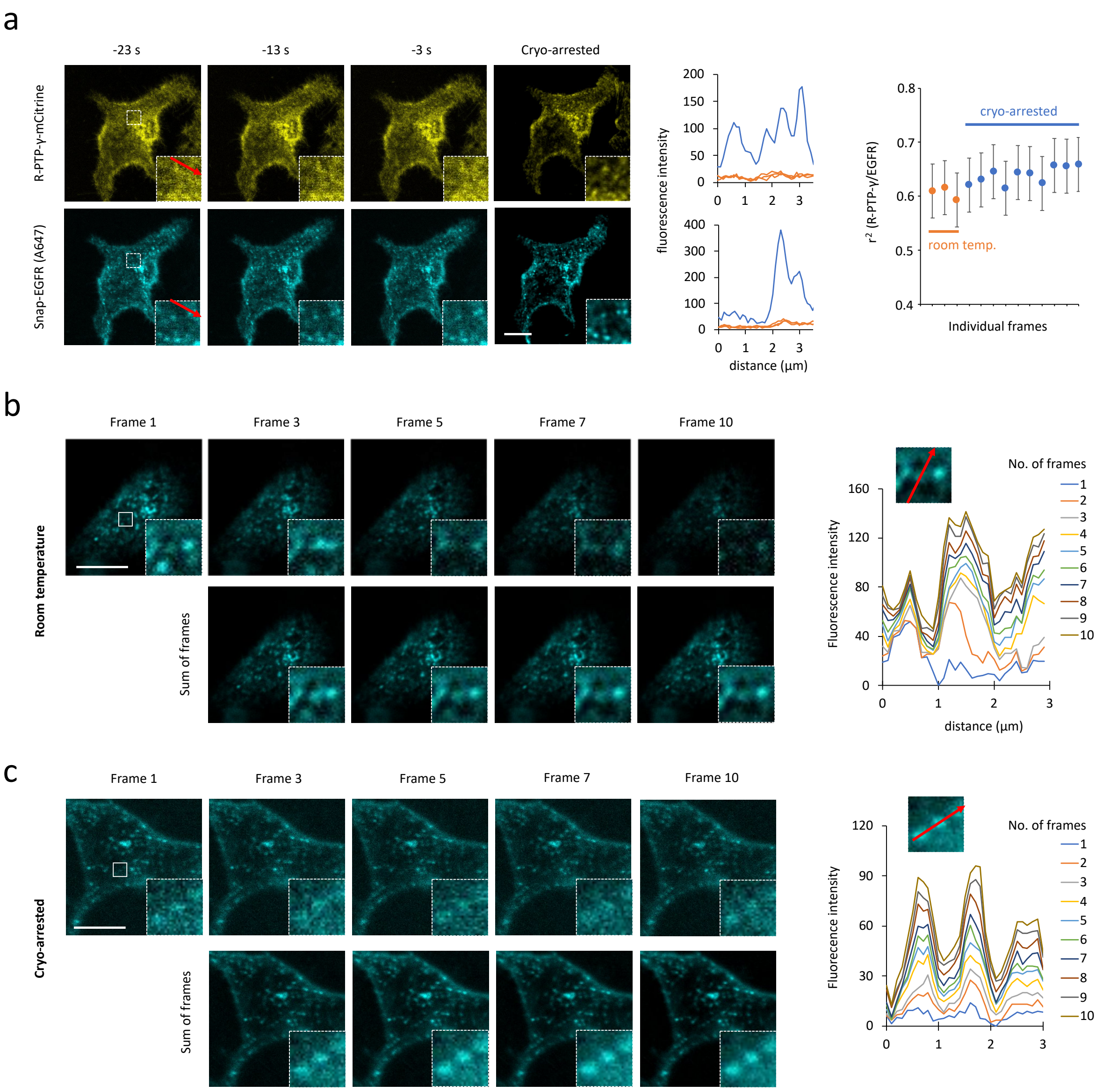

Fig. S3: CLSM of MCF7 cells expressing R-PTP-γ-mCitrine and Snap-EGFR

a) Dual-colour CLSM images of R-PTP-γ-mCitrine (yellow) and Alexa647-labelled Snap-EGFR (cyan) in MCF7 cells before (at indicated time, 3.75 s scan time) and during cryo-arrest (10 frames, 37.5 s). Inserts: Magnification (3x) of the boxed areas. Graphs: Left: Fluorescence intensity profiles before (orange lines) and under cryo-arrest (blue) along lines depicted in the first inserts; right: Coefficient of determination (r^2) between Alexa647-Snap-EGFR and R-PTP-γ-mCitrine on individual frames before (orange dots) and during (blue) cryo-arrest (error bars: uncertainty determined by warping the image). **b-c)** 5 out of a total of 10 recorded frames of MCF7 cells expressing Snap-EGFR imaged at room temperature **(b)** and under cryo-arrest **(c)**. Upper rows: individual frames; Lower rows: sum of fluorescence. Inserts: magnifications of the boxed areas. Graphs: Accumulated, background corrected intensities of the line profiles along the red arrows depicted in the inserts. All scale bars: 10 µm.

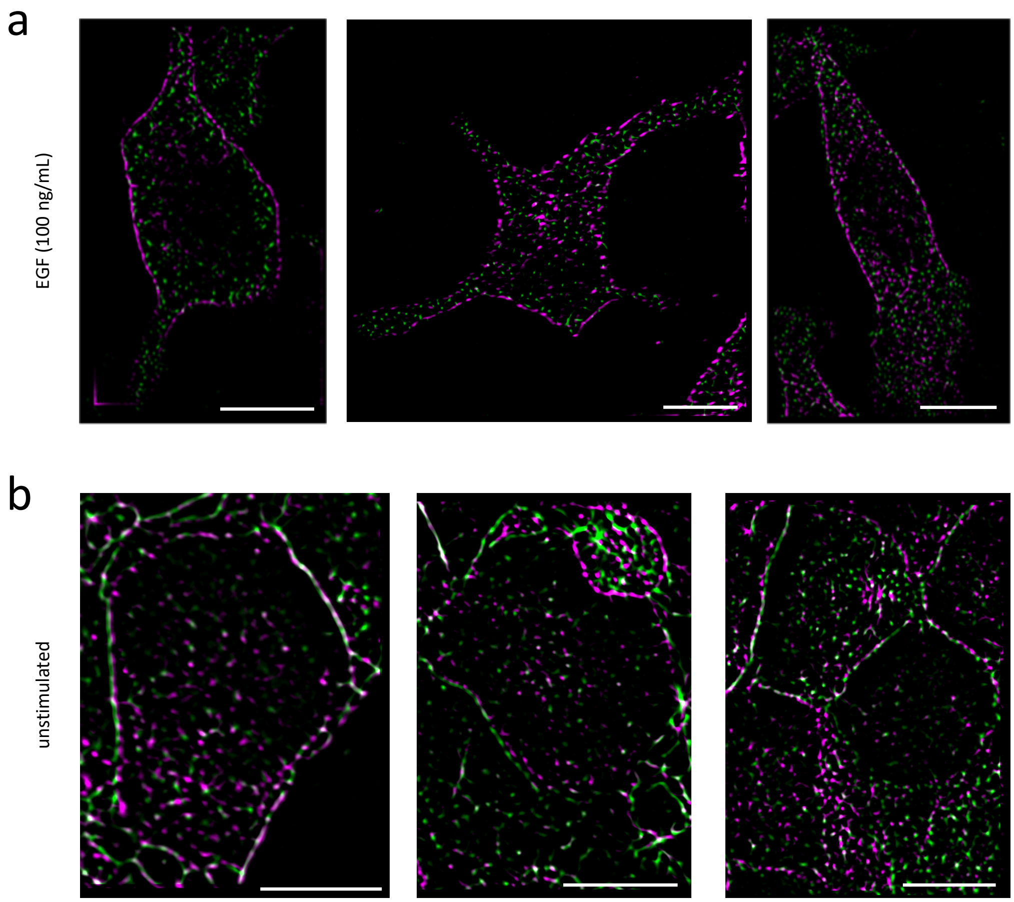

Fig. S4: SRRF reconstructions of R-PTP-γ-mCitrine and Alexa647-Snap-EGFR in MCF7 cells
Representative dual-colour SRRF images of R-PTP-γ-mCitrine (magenta) and Alexa647-labelled Snap-EGFR (green) in MCF7 cells cryo-arrested 15 min after 100 ng/mL EGF stimulation **(a)** or without stimulation **(b)**. SRRF images were reconstructed from series of 100 widefield images per channel. All scale bars: 10 μm.

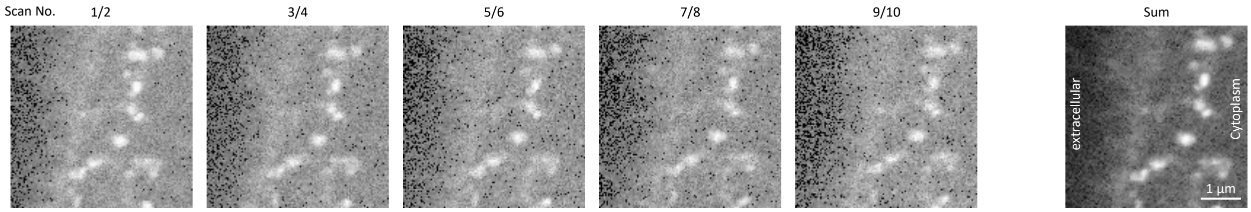

Fig. S5: STED reveals no evidence of nanoscopic ice crystals after ultra-rapid cryo-arrest.
Representative series of 10 consecutive STED scans of a 5 x 5 µm area of Alexa647-Snap-EGFR in EGF-stimulated (100 ng/ml) MCF7 cells under cryo-arrest. Images are shown as sum of 2 scans and gamma corrected (γ: 0.24) to highlight dim fluorescence. The plasma membrane with protrusions is visible as a continuous fluorescence area separating cytoplasmic fluorescence with endocytic structures from extracellular autofluorescence (extracellular). The series was acquired at the maximum laser intensity (0.42 W/μm^2). No ice crystals of 80 nm (digital resolution) or larger, which would exclude fluorophores, scatter the light and appear as dark spots (compare Fig S1b), were detected in the integrated image nor did they form during scanning due to possible heating by the STED beam.

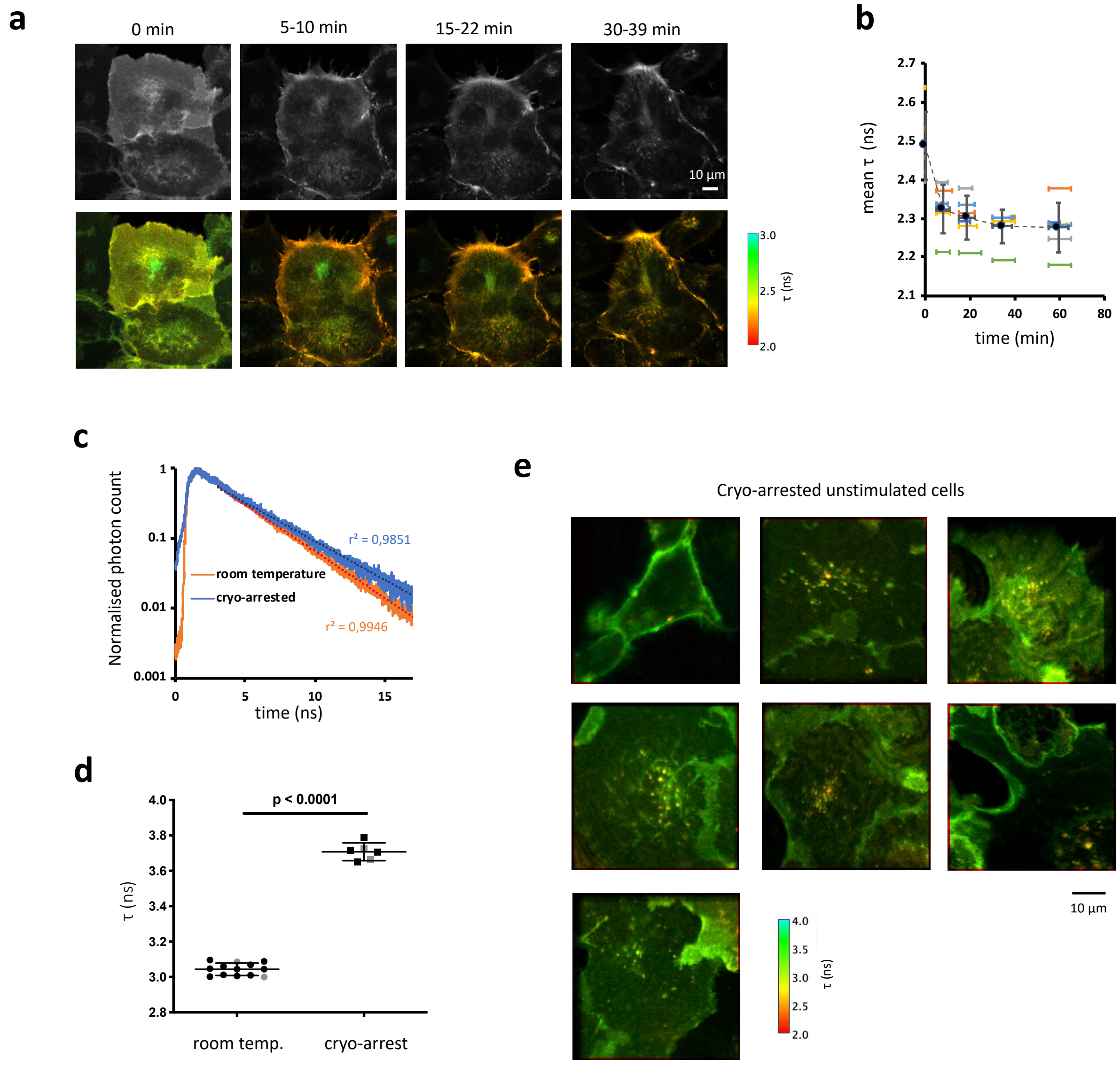

Fig. S6: Measurement of reaction states using FRET-FLIM

a) Confocal FLIM of LIFEA2-expressing Cos7 cells after indicated times of clustered EphrinA1 stimulation measured at room temperature. Upper row: Fluorescence intensity; Lower row: intensity-weighted average fluorescence lifetime (τ); Colour bar: τ (ns). **b)** Mean τ per experiment. Horizontal length of the bars: acquisition time. Individual measurements are colour-coded, Black: mean +/- s.d., N=6 **c)** Exemplified fluorescence decay histograms of MCF7 cells expressing the fluorescent protein mCitrine fused to TC-PTP recorded at room temperature (orange line) and under cryo-arrest (blue); Black dotted lines: mono-exponential tail fits with depicted coefficients of determination (r^2). **d)** τ of mCitrine fused to R-PTP-γ (N=2, grey symbols: single cell data) or TC-PTP (N=2, black symbols: single cell data) expressed in MCF7 cells; mean +/- s.d. **d)** Intensity-weighted τ images obtained by confocal FLIM under cryo-arrest of unstimulated Cos7 cells stably expressing LIFEA2; Colour bar: τ (ns)
